# Inflammatory Cytokine Profiles During Exercise in Obese, Diabetic, and Healthy Children

**DOI:** 10.4274/jcrpe.v3i3.23

**Published:** 2011-09-09

**Authors:** Pietro R. Galassetti, Jaime S. Rosa, Shirin Heydari, Stacy R. Oliver, Rebecca L. Flores, Andria M. Pontello, Milagros Ibardolaza

**Affiliations:** 1 University of California, Department of Pharmacology, Irvine, CA, USA; 2 University of California, Institute for Clinical and Translational Science, Irvine, CA, USA; +1 714 456 53 57 pgalasse@uci.edu

**Keywords:** Interleukin-6, tumor necrosis factor-a, interleukin-2, chemokines, exercise, T1DM, pediatric obesity

## Abstract

**Objective:** Modulation of inflammatory status is considered a key component of the overall health effects of exercise.  This may be especially relevant in children with obesity (Ob) or type 1 diabetes (T1DM), in which an imbalance between pro- and anti-inflammatory mediators could accelerate onset and progression of cardiovascular complications. To date, exercise-induced alterations in immuno-modulatory mediators in Ob and T1DM children remain largely unknown.

**Methods:** In this study, we monitored the kinetic profiles of 8 pro-and anti-inflammatory cytokines  (TNF-a, IL-6, IL-2, IL-8, IL-5, IL-13, IL-10, IL-4) during a standardized exercise challenge (ten 2-min cycling bouts at 80% VO2max, separated by 1-min intervals) in 23 Ob (12 females, 11 males),  23 T1DM (10 females and 13 males) patients and 20 healthy (CL, 10 females and 10 males) children. Blood glucose of T1DM patients was kept in the 4.4-6.1 mM range for at least 90 minute prior to and during exercise.  Blood samples were drawn at rest and after every other exercise bout.

**Results:** In Ob, TNF-a and  IL-2 were significantly greater (p<0.0167) as compared to T1DM and CL, both at baseline and throughout exercise.  All other variables, while not significant, were quantitatively elevated in Ob vs. CL. In T1DM, IL-4 and IL-8 levels were similar to Ob, IL-2 and TNF-a  similar to CL, and IL-6, IL-5, IL-13, IL-4 levels were intermediate between the Ob and CL groups.

**Conclusions:** During exercise, therefore, both Ob and T1DM children displayed exaggerated pro-inflammatory responses, although with clearly different magnitude and involved mediators. Our data support the necessity to identify specific exercise formats through which each at-risk pediatric population can draw maximal beneficial health effects.

**Conflict of interest:**None declared.

## INTRODUCTION

**Introduction **

Incidence and prevalence of common dysmetabolic states, such as obesity (Ob) and diabetes, are rapidly increasing in western societies, most alarmingly among children and adolescents (1,2,3), and are associated with an increased risk of long-term cardiovascular complications (4,5,6).  While the biochemical link between Ob, diabetes and future cardiovascular event is not completely elucidated, a chronic increase in inflammatory processes is now considered one of the main underlying patho-physiological mechanisms (7). 

 Physical exercise has been empirically known for decades to exert long-term protection against cardiovascular disease (8,9). More recently, this protective effect has been linked to  exercise-induced modulation of inflammatory processes (10,11,12).  Acutely, i.e. in response to a single bout of exercise, a pro-inflammatory response is generated (increase in circulating leukocytes and in systemic concentrations of pro-inflammatory cytokines and chemokines), whose damaging potential is limited by simultaneous activation of anti-inflammatory mechanisms (13,14).  Conversely, repeated exercise training results in significant reduction of the systemic inflammatory state (12,15).  The overall health effects of exercise are therefore induced by the correct balance between these apparently opposed pro- and anti-inflammatory effects (16). Our overarching hypothesis is that in conditions characterized by a state of chronically increased inflammation (such as Ob and diabetes) this balance may become altered and the beneficial effects of at least some formats of exercise may be reduced, or, in extreme cases, eliminated (17).  Consequently, gaining a thorough understanding of all biochemical details associated with inflammatory regulation during exercise and of alterations, if any, in these biochemical parameters in states of Ob and diabetes, appears as a necessary pre-requisite for the design and implementation of effective preventive and/or treatment strategies based on exercise interventions.  

Most available data in this field have been obtained from studies in adults; extrapolation of these results to children is conceptually inaccurate, as most metabolic and hormonal responses differ substantially between these age groups (18).  Further, even in adults, most studies evaluating inflammatory responses to exercise report measurements before, at the end, and sometimes after the exercise challenge, largely ignoring the time-course of these changes during exercise.  To fill an important knowledge gap in this area, we have therefore designed the current study to evaluate concentrations and time course of a panel of key inflammatory mediators during a standardized exercise challenge reproducing real-life physical activity.  The study was performed in CL children and in children with the two most common pediatric dysmetabolic conditions, Ob and type 1 diabetes (T1DM).  

## METHODS

**Subjects and Preliminary Visit**

 This study was conducted at the UCI ICTS (University of California, Irvine Institute for Clinical and Translational Science) following UCI IRB (Institutional Review Board) approval.  Participants were divided in three groups: Ob, n=23 (12.5±0.5 years, 12 females), T1DM, n=23 (13.9±0.3 years, 10 females), and CL, n=20 (12.9±0.9 years, 10 females) (Table 1). 

At a preliminary visit, all participants completed a  questionnaire regarding their pubertal status and the information was assessed using the Tanner stages (19). Inclusion in the Ob group was done in accordance with the current criteria set by the Centers for Disease Control and Prevention (CDC), i.e., an individual age- and gender-adjusted BMI of  ≥95th percentile.  Children in the T1DM and CL groups had a BMI %<85th percentile.

A ramp exercise test on a braked cycle ergometer (SensorMedics Ergoline 800S, Yorba Linda, CA) was then conducted to assess each individual’s cardiopulmonary fitness.  Work-rate was increased ˜10W/min for roughly 12-14 min until maximal tolerated workload was reached. Maximal oxygen uptake [VO2max, or the gold standard assessment of physical fitness, and anaerobic threshold (AT), or the point at which additional skeletal muscular work must be accompanied by anaerobic biochemical mechanisms] (20) were measured by gas exchange using a standard metabolic cart (SensorMedics VMax 229, Yorba Linda, CA).  

**Main Study Day & Collection of Blood Samples**

On study day, the subjects were admitted to UCI ICTS at 7 AM after an overnight fast. Upon admission, vital signs were checked and an intravenous catheter was inserted  into the right arm for IV infusion (insulin/dextrose for T1DM and saline for Ob and CL subjects); a second catheter was placed into the left arm to draw blood samples. CL and Ob subjects then rested for 90 minutes in order to minimize the effect of venipuncture stress on measured variables, and then performed a standardized exercise challenge as described below. 

**Figure 1:**
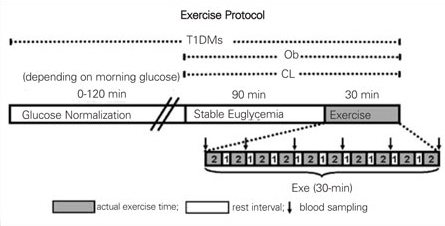
Figure 1. Experimental design Subjects from all 3 groups rested for 90 min in euglycemic range after establishment of intravenous line (T1DM required insulin and glucose infusions to achieve euglycemia prior to the 90-min period and euglycemic clamp throughout the study).  The exercise paradigm consisted of 2-min cycling at ˜80% VO2max followed by 1-min rest, completed 10 times (30 min total, 20 min of actual cycling and 10 min rest in between).  Blood samples were drawn immediately before and during (every 6 min) exercise.  White bar denotes resting period; grey bar, cycling; black arrow, blood drawn for cytokine measurements

For T1DM patients whose glucose levels upon admission were outside the euglycemic range, glycemia was normalized via IV insulin infusion and maintained between 4.4-6.1mM for 90 minutes prior to the start of exercise. All subjects then performed a 30-min intermittent exercise test that consisted of ten, 2-minute bouts of constant work on a cycle ergometer with 1-minute intervals of rest between each bout (therefore, a total of 20 min of actual cycling and 10 min of rest).  Work-rate was adjusted to each individual, so that it was at 50% of the difference between the anaerobic threshold and the VO2max, or ˜80% VO2max. This procedure normalized the level of exercise intensity to each participant’s exercise capacity. Blood samples were drawn immediately prior to exercise start, after every other bout (5,6,10,12) and at end-exercise to analyze cytokines and plasma glucose fluctuations (Figure 1).

**Laboratory Measurements**

At all study time points, a 1 mL blood sample was spun in a sterile heparin-coated microcentrifuge tube, and plasma glucose was measured with a Beckman Coulter Glucose II Analyzer (Beckman Coulter, Fullerton, CA).  Following glucose measurement, the remaining plasma volume was frozen at -800C until the day of cytokine assay.  Samples were slowly brought to room temperature on the day of experimental run and immunoassayed using LINCOplex microbeads (LINCO Research, St. Charles, MO; catalog # HSCYTO-60SK; intra-assay %CV 3.11-5.86, intra-assay %CV 2.16-14.27).  The following day, a conjugated bead immunoassay was performed on a Luminex 200 analyzer (Luminex Corp., Austin, TX) utilizing the MasterPlex Software (Miraibio, Alameda, CA).   

**Statistical Analysis**

Demographic and circulating inflammatory markers data of the three groups (Ob, T1DM, CL) were expressed as group means±standard error (SE). The mixed model was used to detect changes in measured variables during exercise over time; area under the curve (AUC) was used to assess differences in variables across groups during  exercise.  Statistical significance for baseline and AUC values was determined using unpaired, two-tailed Student’s t-test, with Bonferroni correction. All statistical analyses were  performed using JMP software (SAS Institute, Cary, NC). A p<0.0167 level after correction was considered as significant.

**Table 1:**
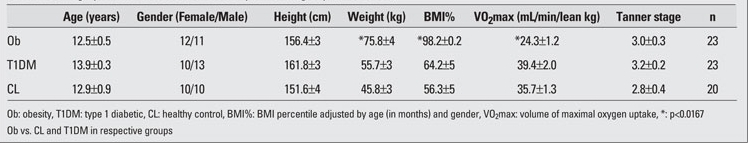
Table 1. Demographic characteristics of the 3 experimental groups (means±SE)

## RESULTS

**Subjects**

There was no difference across the three groups in terms of age, gender distribution, height, weight, and Tanner stage: As expected, the Ob group had significantly greater weight, and BMI%; consequently, VO2max, expressed per total body mass, was also reduced in this group.  If expressed per lean body mass, however, VO2max was similar across groups (Table 1).   

**Inflammatory Mediators**

**Figure 2:**
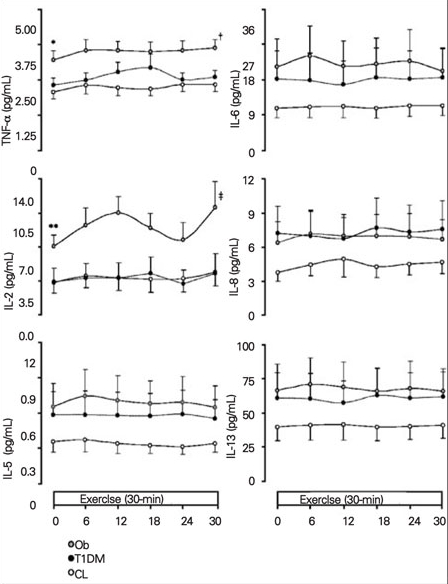
Figure 2. Elevated levels of pro-inflammatory markers in response to exercise for the 3 experimental groups Levels of tumor necrosis factor-a (TNF-a) and IL-2  in obesity (Ob) (grey circle), type 1 diabetic (T1DM) (black circle), and healthy control (CL) (white circle) groups at baseline and during exercise. Data are expressed as means±SE in the 3 groups, Ob vs. CL at baseline *(p<0.0167); Ĵ(p<0.0167), AUC: Ob vs. CL; ** (p<0.0167), Ob vs. CL and T1DM; ĵ(p<0.0167), AUC: Ob vs. CL and T1DM

Our results followed a relatively stable pattern. While the standardized exercise challenge did not illicit an increased inflammatory response in any of the groups, the Ob children displayed the greatest cytokine values, and the CL children the lowest (with only some of these differences being  statistically significant) throughout exercise.  T1DM children were somewhat in the middle, with values similar to the CL group in 3/8 variables, and with some degree of elevation in the remaining cases (Figure 2). 

**• Interleukin-6**

Throughout exercise, in the Ob group, IL-6 mean  concentrations were quantitatively greater (range 20.5-24.3 pg/mL) than in the control group (range 10.9-11.7 pg/mL).   The mean level of IL-6 for the T1DM group (range 17.0-18.8 pg/mL) stayed in the intermediate range between the Ob and CL groups.   

**• Tumor Necrosis Factor-a (TNF-a) and Interleukin-2**

In the Ob group, TNF-a was significantly greater (range  3.2-3.6 pg/mL) than in the CL and T1DM children (ranging from 2.1-2.3 pg/mL, 2.2-2.6, respectively, p<0.0167), and remained significantly elevated throughout exercise (AUC: Ob, 84.6±7.5; CL, 54.1±5.6; T1DM, 57.8±6.2,  p<0.0167). For IL-2, the Ob group had significantly higher values (range 7.1-11.1 pg/mL) than the T1DM (range 3.2-4.3 pg/mL) and CL (range 3.3-4.4 pg/mL) groups (p<0.0167).  

**• Other Pro-Inflammatory Mediators (IL-5, IL-8, IL-13)**

In the Ob and T1DM groups, the levels of IL-5, IL-8, and  IL-13, pro-inflammatory mediators were elevated as compared to the CL group, albeit no significance was  detected.  For each experimental group, there was  consistency from baseline throughout exercise for IL-5 and IL-13, in that the amount of oxidative stress markers that were released were shown to be higher in the Ob children compared to the T1DM and CL subjects.  For IL-5, levels in the Ob group stayed between 0.6-0.7 pg/mL, whereas those in the T1DM and CL groups were between 0.2-0.5 pg/mL.  As for IL-8, the Ob group expressed levels between 6.4-7.7 pg/mL, very similar to those of the T1DM group.  However, it is important to note that the CL group showed lower values in all three assays of these cytokines.  

**• Anti-Inflammatory Mediators (IL-10 and IL-4)**

**Figure 3:**
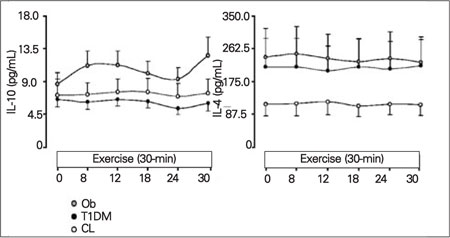
Figure 3. Elevated levels of anti-inflammatory markers in response to exercise for the 3 experimental groups Levels of anti-inflammatory cytokines, IL-10 and IL-14 in obesity (Ob) (grey circle) , type 1 diabetic (T1DM) (black circle), and healthy control (CL) (white circle) groups at baseline and during exercise.  Data are expressed as means±SE

The pattern observed for IL-10 showed elevated values in the Ob group. Values in T1DM subjects were about the same as CLs.  The Ob group fluctuated between 8.7-12.7 pg/mL and the T1DM and CL were between 5.4-7.6 pg/mL.  However, as to IL-4, cytokine levels were elevated in the Ob and T1DM groups as opposed to the CLs. The levels in the CL group stayed low between 110.6-122.1 pg/mL, whereas those in the T1DM and Ob groups were between 203.5-249.2 pg/mL. No significant differences were detected among the groups in  anti-inflammatory mediator values (Figure 3).

**Table 1:**
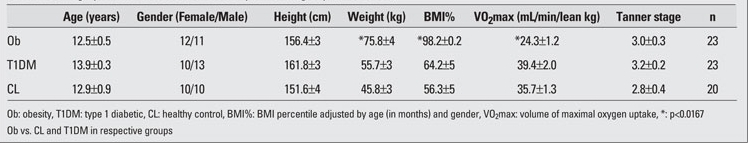
Table 1. Demographic characteristics of the 3 experimental groups (means±SE)

## DISCUSSION

Using a conjugated micro-bead immunoassay and an exercise model representative of daily physical activity, we identified a number of differences in inflammatory mediator concentrations between CL, Ob and T1DM children.  All 8 tested cytokines were quantitatively elevated in the Ob group as compared to CLs, reaching statistical significance for TNF-a and IL-2. T1DM subjects also displayed some degree of elevation, which was, however, as marked as in the Ob group only for IL-5 and IL-8; for the other measured variables, the elevation was either modest or, in the case of TNF-a and IL-2, completely absent.  While some prior studies had focused on similar issues, information had been fragmented by the fact that individual variables and subject groups were studied separately and with different protocols, rendering quantitative comparison problematic. To our knowledge, ours is the first comprehensive report of a broad cytokine panel tested simultaneously in children, during a standardized exercise challenge, in these three conditions. 

Our results are consistent with prior reports of elevated inflammatory status in these patient groups. Several authors reported a positive correlation between Ob/visceral adiposity and several inflammatory markers, including TNF-a, IL-6, and C-reactive protein, both in adults (21) and children (22,23).  Conversely, a significant decrease in systemic levels of inflammatory markers were reported in Ob subjects after weight loss (24,25). Further, in young T1DM subjects, Erbagci et al (26), among others, reported elevated TNF-a and IL-8 in newly diagnosed (<1year) patients, and elevated IL-2 levels in all T1DM subjects when compared to age- and gender-matched CLs.  Several previous studies from our laboratory have also confirmed the presence of elevated levels of IL-6 in T1DM children, relative to CLs, both at rest and during exercise (27), and correlated the degree of the IL-6 elevation with the magnitude of hyperglycemic episodes to which subjects had been exposed during the previous hours and days (28,29). Taken together, and considering the now well-established link existing between chronically increased inflammatory status and long-term development of cardiovascular complications, our observations in Ob and T1DM subjects indicate that some of the pathogenetic mechanisms leading to these complications may be already active at the very early stages of these conditions. 

While in general inflammatory status appeared increased in both our groups of patients, the pattern of elevation of individual inflammatory mediators was clearly different between T1DM and Ob groups, with the latter group displaying an overall considerably greater level of activation of both pro- and anti-inflammatory cytokines.  The existence of such a difference did not come as a surprise. Inflammatory homeostasis encompasses an extremely complex series of processes, regulated by hundreds, possibly thousands of different components, only some of which may be specifically activated in response to individual stimuli 30,31,32,33,34). In Ob, for instance, while occasional exacerbations may be induced by acute hyperlipidemia or other overlapping pathologies, a chronic, subclinical but pro-inflammatory stimulus is provided by the adipose tissue itself, within which a large number of activated macrophages continuously secrete chemokines and cytokines (35). On the other hand, in T1DM it is still debated whether presence of the disease per se implies pro-inflammatory activation (36). In these patients, however, acute, repeated and frequent pro-inflammatory signals are created with each hyperglycemic episode (37,38), the magnitude, duration and repetition pattern of which may determine the degree and type of involved inflammatory process (28,39). In these patients, as in the group of T1DM subjects in our study, prior hyperglycemia had not been brought under control, and it is likely that a mixed level of pro-inflammatory activation existed across subjects, possibly resulting in the less pronounced alterations that were observed as compared to the Ob group.  It is therefore possible, although it would have to be confirmed with a separate set of studies, to speculate that T1DM recently exposed to severe hypoglycemia would display an inflammatory response as pronounced, if not even greater, than that observed in Ob children. Differences across subject groups may also have been amplified by the fact that exercise causes an independent, albeit physiological, rise in inflammation, if superimposed to an already enhanced inflammatory milieu (such as in the Ob children), and that this  may have unevenly enhanced activation of some of its components. 

Central to this study was the exercise protocol, which was specifically developed in our laboratory for use in children, and has now been applied thousands of times in a variety of experimental settings and different study populations. The controlled exercise work-rate (˜ 80% of VO2max, a definitely intense level of exertion), and the 1-minute breaks in between each 2-minute bout were meant to reproduce as much as possible a real-life exercise challenge (e.g. a soccer or basketball practice, or a dance session) while eliciting a strong and widespread cytokine response.  Cycle ergometry was chosen over treadmill running due to the closest correlation obtainable with the former technique between selected workload and work actually performed by the subject. It also important to note that for exercise responses to be meaningfully compared across different experimental groups, the level of fitness should be comparable across study participants.  In our study, CLs and T1DM patients indeed displayed similar VO2max/kg values, while in the Ob group, these were substantially lower (Table 1) due to their greater body weight.  If normalized by lean body mass, however, which is a better predictor of exercise capacity than total body weight, VO2max was similar across all three groups.  The fact that the relative intensity of the exercise challenge was the same in all groups was also testified by a similar exercise-induced increase in plasma lactate. A separate set of issues concerned our T1DM subjects, for whom optimal glycemic control before and during exercise was an indispensable prerequisite to data interpretation (had they exercised in hyperglycemic conditions, in fact, it would have been impossible to determine what would have caused the elevated cytokine levels).   As stated in the Methods section, we therefore included in the study design a stabilization period of a minimum of 90-minute before exercise, in which plasma glucose had to be in the normal range. If a T1DM patient displayed elevated glucose levels at admission, insulin was infused intravenously, plasma glucose was checked repeatedly until it reached the normal range, (80-110 mg/dL), which in some instances took up to two hours; then the 90 min of stabilization was started, and exercise was performed only after all this was completed.  Applying these steps in methodology, our laboratory has achieved, safely and effectively, stable euglycemic conditions before, during and after exercise in hundreds of human studies over the last decade (23,40,41,42,43).  

The authors are aware that the choice of using a conjugated micro-bead assay to determine cytokine concentrations, as opposed to the standard ELISA technique, may be prone to controversy, as results obtained with this methodology were at times found to vary in absolute values when matched with gold-standard techniques. Other studies, however, reported very good correlations (44), and our laboratory has independently validated these results by running matching ELISA tests on a subset of study samples, with excellent agreement. Further demonstration of the quality of our data was the fact that within the multiple time points obtained from each subject, the data was very consistent.  Also, considering that the study subjects were children, a definitely vulnerable population, the volume of total blood drawn was an issue, and the ability of the assay to simultaneously measure a large number of values in a single sample of a volume of a fraction of 1 mL rendered the conjugated micro-bead assay especially well suited for our experiments. It should also be noted that, to further minimize the potential for variability, all assays were performed at the same time and using materials from the same company.

We believe that results from our study may have implications for the design and implementations of specific exercise guidelines in Ob and diabetes. While it is relatively well established that exercise provides long-term cardiovascular protection, and that modulation of inflammatory processes likely plays a key role in this effect, the biochemical details of these interactions remain undefined. Stating that “inflammation is increased” in Ob and diabetes is obviously not enough to provide workable conceptual bases for specific exercise strategies (45). Our study demonstrates that different components of the inflammatory cascade may be activated in different groups of subject.  We can safely assume that additional differences may exist for yet other common chronic conditions, as well as with use of exercise formats of different type, intensity, duration, or repetition pattern. The next logical steps therefore appear to be first identifying all individual inflammatory mediators involved in each exercise format, and then the possible alterations of their activation pattern induced by specific pathological conditions.  Only then we will be able to truly develop optimal exercise regimens, differently calibrated for narrowly focused groups of subjects.  We believe our results represent a small step in this direction. 

In summary, we are reporting the observation that circulating concentrations of pro- and anti-inflammatory cytokines were greater in Ob children, and to a lesser extent in T1DM patients, as compared to CLs.  As physical exercise is advocated as a key management strategy in these conditions, but chronic inflammation is also considered a leading cause of their long-term cardiovascular complications, our results raise the issue of what exercise type and formats should be implemented to optimize health benefits in situations characterized by a chronically elevated, subclinical inflammatory status. 

**Acknowledgements**

The authors thank all UCI ICTS nurses and staff for excellent study support, and Brian Tran, Joanne Cheung and Chelsey Azevedo for assistance in the preparation of the manuscript. This study was funded by NIH grants M01-RR00827-28 and K-23 RR018661-01 and Juvenile Diabetes Research Foundation grant #11-2003-332.

**Table 1:**
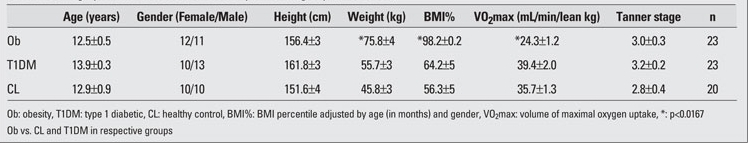
Table 1. Demographic characteristics of the 3 experimental groups (means±SE)
